# Recording of Bipolar Multichannel ECGs by a Smartwatch: Modern ECG Diagnostic 100 Years after Einthoven

**DOI:** 10.3390/s19132894

**Published:** 2019-06-30

**Authors:** Alexander Samol, Kristina Bischof, Blerim Luani, Dan Pascut, Marcus Wiemer, Sven Kaese

**Affiliations:** Department of Cardiology and Critical Care Medicine, Johannes Wesling University Hospital, Ruhr University Bochum, 32429 Minden, Germany

**Keywords:** electrocardiogram (ECG), smartwatch, Einthoven, single-lead ECG, multichannel ECG

## Abstract

*Aims:* Feasibility study of accurate three lead ECG recording (Einthoven I, II and III) using an Apple Watch Series 4. *Methods*: In 50 healthy subjects (18 male; age: 40 ± 12 years) without known cardiac disorders, a 12-lead ECG and three bipolar ECGs, corresponding to Einthoven leads I, II and III were recorded using an Apple Watch Series 4. Einthoven I was recorded with the watch on the left wrist and the right index finger on the crown, Einthoven II with the watch on the left lower abdomen and the right index finger on the crown, Einthoven III with the watch on the left lower abdomen and the left index finger on the crown. Four experienced cardiologists were independently asked to assign the watch ECGs to Einthoven leads from 12-lead ECG for each subject. *Results:* All watch ECGs showed an adequate signal quality with 134 ECGs of good (89%) and 16 of moderate signal quality (11%). Ninety-one percent of all watch ECGs were assigned correctly to corresponding leads from 12-lead ECG. Thirty-nine subjects (78%) were assigned correctly by all cardiologists. All assignment errors occurred in patients with similar morphologies and amplitudes in at least two of the three recorded leads. Erroneous assignment of all watch ECGs to leads from standard ECG occurred in no patient. *Conclusion:* Recording of Einthoven leads I-III by a smartwatch is accurate and highly comparable to standard ECG. This might contribute to an earlier detection of cardiac disorders, which are associated with repolarization abnormalities or arrhythmias.

## 1. Introduction

Smartwatches or other smart medical devices show an enormous increasing use in Western countries. Nearly 77% and 13% of the US citizens are actually owner of a smartphone and/or a smartwatch, respectively [1]. Older generations of smartwatches like the Apple Watch Series 3 ^®^ (Apple Inc., Cupertino, CA, USA) allow recording of pulse frequency and irregularities using photoplethysmography with LED lights and light-sensitive photodiodes located on the backside of the watch [1]. This tool seems to be useful in detection of arrhythmias with high sensitivity and specificity [2]. Portable electrocardiogram (ECG) devices or handheld electrocardiogram devices like the AliveCor (AliveCor Inc., Mountain View, CA, USA) or MyDiagnostick (Applied Biomedical Systems BV, Maastricht, The Netherlands) for smartphones allow patient-activated recording of electrocardiograms, but carrying along the ECG device is required for ECG documentation [3,4,5,6].

The US version of the new Apple Watch Series 4 ^®^ (Apple Inc, Cupertino, CA, USA) allows recording a single-lead ECG [7].The bipolar ECG-lead is derived by recording of the voltage difference over time between the right index finger and the left arm wrist and simulates Einthoven’s ECG lead I. ECG recording is activated by the patient, and thereafter, a pdf document of the ECG is created by using the Apple Health App which can be printed or sent to the doctor. Nevertheless, discriminating P-waves in only one lead for diagnosis of sinus rhythm is sometimes challenging [8] and often requires recording of more than one ECG lead for sufficient detection of the P wave [4] In addition, recording of more than one ECG lead is required for cardiac disorders such as myocardial infarction, pulmonary embolism or acute left and/or right heart failure.

In 1903 Einthoven developed recording of a bipolar three channel ECG [9]. This three lead ECG enables identification of the electrical heart axis using the Einthoven’s triangle and might identify acute or expired ischemia, especially in inferior regions of the myocardium [10]. Today, Einthoven leads are an integral part of the standard 12-lead ECG in daily clinical practise and are required for e.g., determination of heart rhythm or myocardial ischemia.

Diagnosis of myocardial infarction (MI) has been improved in the last decades. However, there is still delayed diagnosis in a not negligible number of patients, especially if patients hesitate to contact a physician or the emergency medical service immediately after onset of symptoms, which may lead to a delay in initiation of adequate treatment and thus prolongs the time interval to first medical contact [11]. Recording and sending a multichannel ECG by a wearable device might shorten the time to first medical contact, contributing to an earlier treatment and a better outcome of the patients [6].

Several studies have used smartphones, smartwatches or handheld electrocardiogram devices in cardiology patients, mainly for detection of atrial fibrillation (AF) [1,2,4,8]. The Apple Heart Study used photoplethysmography of the Apple watch for identification of pulse irregularity or variability, which may be used for detection of AF [1]. A similar study, the WATCH AF trial also used a smartwatch-based algorithm with photoplethysmographic signals for AF detection and demonstrated that AF was recorded with very high diagnostic accuracy [2]. A limitation of the algorithm was a high dropout rate based on insufficient signal quality [2]. The study by Tison et al. also demonstrated that smartwatch photoplethysmography was able to detect AF but also found reduced sensitivity and specificity compared to the standard ECG [12].

A limitation of photoplethysmography is that pulse irregularities are used for detection of AF. As supraventricular and ventricular extra systoles may also cause pulse irregularities, AF detection may be impeded by photoplethysmography. Therefore, recording of an ECG by a smartphone or smartwatch watch may increase diagnostic accuracy. Several handheld electrocardiogram devices like the AliveCor (AliveCor Inc., USA) or MyDiagnostick (Applied Biomedical Systems BV, The Netherlands) are available for smartphone and allow recording of a single-lead ECG, which is Einthoven lead I [4,8]. The AliveCor device was used for community AF screening in a study which found a high sensitivity and specificity for AF detection [8]. False positive AF detection was due to small voltage *P* waves in lead I [8]. Another study tested the two handheld ECG devices for AF screening in a hospital population in cardiologic and geriatric patients [4]. In this subgroup of patients, the authors demonstrated that sensitivity and specificity of the automated algorithms were only suboptimal and additional 12-lead ECGs were required to optimize specificity. A limitation of these ECG is that only one ECG lead, Einthoven I, can be recorded, which limits arrhythmia or repolarization detection.

So far, only one study demonstrated in a case series of five patients, that a standard 12 lead ECG could be recorded by the bipolar AliveCor (AliveCor Inc., USA) device, which was modified with standard ECG tabs and wires with alligator clips [3]. ECG recording was feasible with good signal quality but required modification of the device for enabling 12 lead ECG recording is not sufficient outside clinical studies or for patient-directed self-ECG recording. Therefore, we performed a proof of concept and prospective study in healthy voluntary subjects concerning the feasibility and accuracy of recording of a three channel ECG encompassing Einthoven’s leads I, II and III by a common Apple Watch Series 4 in comparison to the same leads recorded by a standard ECG device. To the best of our knowledge this is the first study using a smartwatch for recording of Einthoven leads.

## 2. Materials and Methods

### 2.1. Study Participants

This study was performed according to the Declarations of Helsinki and approved by the Ethics Committee of the Aerztekammer Westfalen-Lippe (reference number 2019-456).

Fifty healthy voluntary subjects (18 male; mean age 40 ± 12 years) with no history of cardiovascular disease were prospectively enrolled in our study.

### 2.2. 12-Lead Surface ECG

A standard 12-lead ECG was recorded using a common ECG device (MAC 5500, GE Healthcare, Chicago, IL, USA) with a paper running speed of 50 mm/s. ECG recording was performed after a resting period of 5 min in supine position.

### 2.3. Smart-Watch ECG Recordings

**Figure 1:**
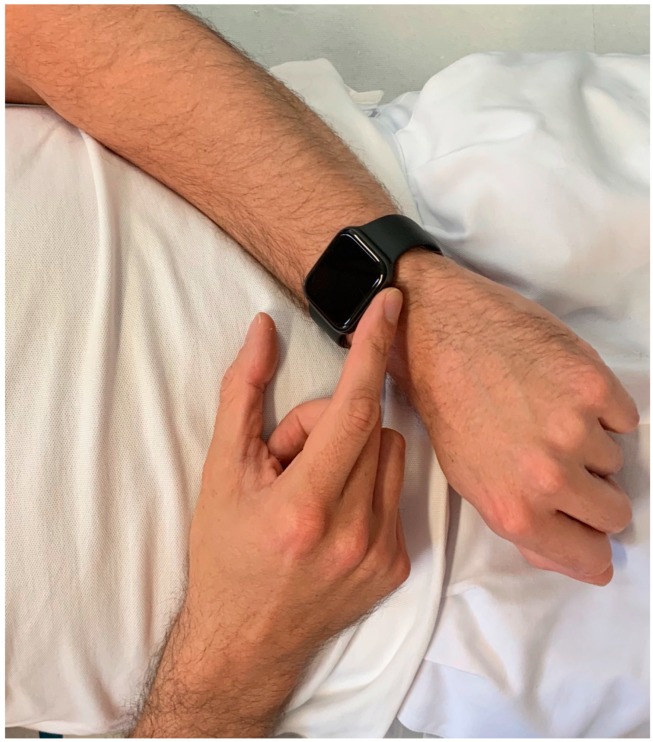
Registration of Einthoven lead I between the left arm wrist and the right index finger.

**Figure 2:**
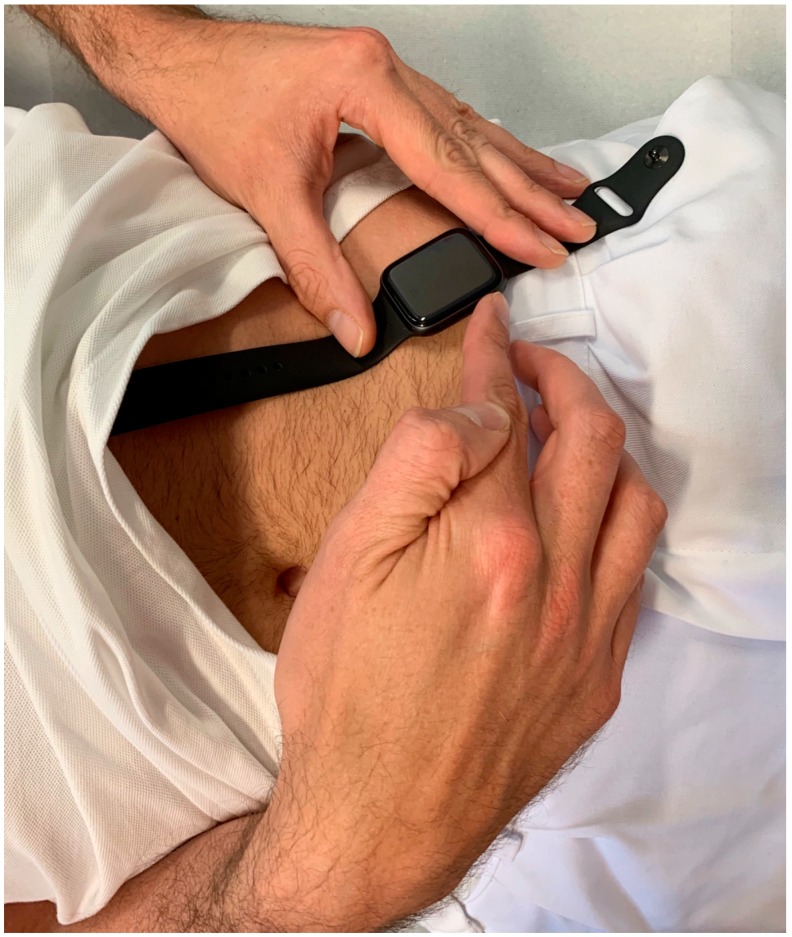
Registration of Einthoven lead II between the left lower abdominal region and the right index finger.

**Figure 3:**
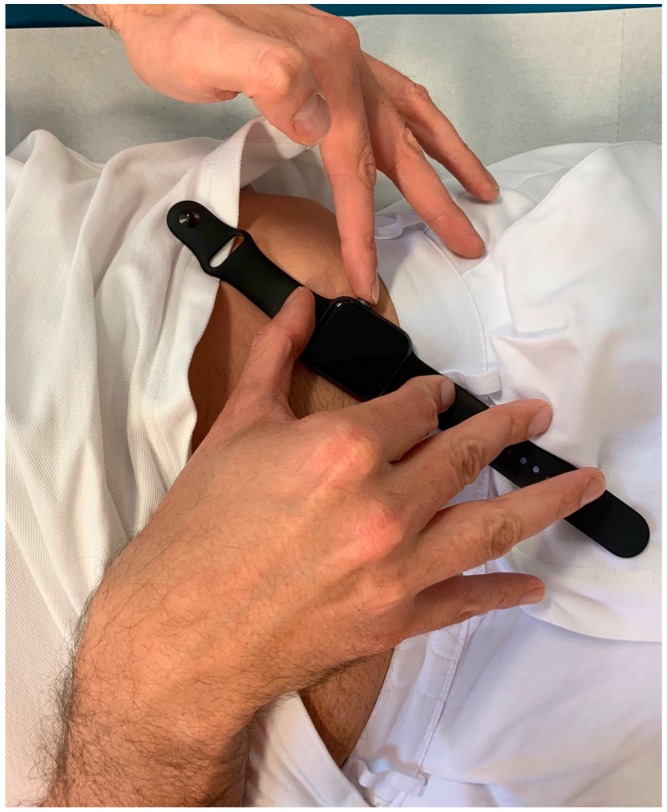
Registration of Einthoven lead III between the left lower abdominal region and the left index finger.

An US model of the Apple Watch Series 4 ^®^ (Apple Inc, Cupertino, CA, USA) was used for ECG recordings immediately after recording the 12-lead ECG. Recording of Einthoven I was performed with the Apple Watch on the left wrist and the right index finger on the crown (Figure 1), Einthoven II was recorded with the watch on the left lower abdomen and the right index finger on the crown (Figure 2), Einthoven III was recorded with the watch on the left lower abdomen and the left index finger on the crown (Figure 3). All recorded ECGs were digitally stored using the Health Application of an iPhone Series 7 (Apple Inc, Cupertino, CA, USA).

Using the “send pdf to your doctor” function, a pdf document of every single ECG lead was created and paper printed for further analysis. All recorded ECGs were classified of moderate signal quality, if at least three consecutive QRS-complexes showed noise-free signal quality inclusive no artifacts in iso-electric lines between QRS-complexes, and of good signal quality if at least ten QRS-complexes showed noise-free signal quality and no artifacts in iso-electric lines between QRS-complexes.

Four experienced cardiologists were independently asked to assign the three Apple Watch ECG recordings to Einthoven I–III leads from the standard 12-lead ECG for each subject. In order to be blinded, the three Apple Watch ECG paper prints were randomly labeled “A”, “B” or “C” for each subject. The cardiologist assigned each single Apple Watch ECG “A–C” by visual comparison to Einthoven’s leads I–III from the standard 12-lead ECG. Thereafter, two not involved physicians using a prepared solution table checked correct assignment.

### 2.4. Statistical Analysis

Statistical analysis was performed using IBM SPSS Statistics (version 24 for Mac, IBM Corporation, Somers, NY, USA). Categorical variables are presented as absolute numbers and percentages. Continuous variables are shown as mean ± standard deviation. Differences of metric outcome variables were assessed by one way repeated analysis of variance (ANOVA) and paired T-Test. In case of binary variables, the χ2-test was used.

## 3. Results

**Figure 4:**
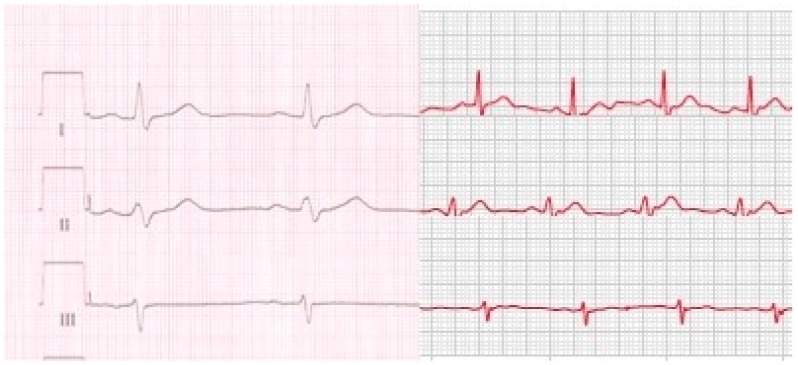
Comparison of typical standard Einthoven I–III leads from standard ECG (black ECG curves) and Apple Watch ECGs I–III (red ECG curves).

Subject characteristics are shown in Table 1. Each of the 50 healthy subjects was able to perform ECG recording by using the Apple Watch after a short instruction. All 150 Apple Watch ECGs showed an adequate signal quality for diagnostics purposes with 134 ECGs of good (89%) and 16 of moderate signal quality (11%). Ninety-one percent of all Apple Watch ECGs were correctly assigned to Einthoven’s lead I, II and III recorded by the standard ECG device by all four cardiologists. Correct assignment of all three ECG leads ranged from 43 to 47 (86% to 94%). Thirty-nine subjects (78%) were correctly assigned by all four cardiologists. Typical ECG recordings are shown in Figure 4. In five of the 11 subjects with at least one incorrect assignment, more than one cardiologist allocated the ECGs incorrectly. All assignment errors were made in patients with comparable morphologies and amplitudes in at least two of the three Einthoven leads recorded by the Apple Watch and/or the standard ECG device. In all these subjects at least one ECG lead was assigned correctly. In these subjects the correctly identified ECG lead was the same throughout the results of all four cardiologists. Subjects with assignment errors were older than the opposite group (Table 2). Subjects’ weight, body height, body mass index (BMI), body surface area (BSA), sex, heart rate and electrical heart axis was not associated with correct assignment (Table 2).

## 4. Discussion

New technologies and modern devices, such as smartphones and smartwatches, have become an integral part of daily life and medical care [1]. These new devices and apps may also raise challenges concerning effective use to promote health and need to be tested in studies [7]. To the best of our knowledge, this is the first study which evaluated feasibility of an Apple Watch Series 4 to record bipolar single-lead ECGs corresponding to the classical Einthoven ECG leads I, II and III.

Each of the 50 healthy subjects was able to perform ECG recordings by themselves in the three required positions by using the Apple Watch corresponding to Einthoven ECG leads I, II and III. This confirms that appropriate use of the smartwatch for multichannel ECG recording is feasible after a short instruction. Of note, the studied healthy subjects were middle-aged and more familiar to modern technologies as compared to older subjects. Appropriate use of a smartwatch for ECG recording by older patients might be less practicable. In accordance, a study using two handheld electrocardiogram devices for recording of a single-lead ECG showed that 7% of cardiology and 21.4% of geriatric patients were not able to sufficiently use the device [4].

The signal quality of all 150 Apple smartwatch ECGs allowed accurate evaluation. Consequently, 91% of all Apple Watch ECGs could be correctly assigned to Einthoven leads I, II and III recorded by the standard ECG device by four independent cardiologists. ECG signal quality with respect to *P*-wave, QRS complex, T-wave and isoelectric line recorded by the Apple Watch seems to be highly comparable to the same recorded by a commercial ECG device.

Therefore, multichannel ECG recordings with smartwatches, which are far more available than common ECG devices and could be recorded by the patients themselves, might accelerate diagnosis of acute cardiac diseases and shorten duration to first medical contact. However, the patient should be reminded that usage of a smartwatch does not replace a doctor’s visit or a standard 12-lead ECG. Other devices for bipolar ECG recording using smartphone technology are available and have also successfully been used for multichannel ECG recordings [3]. Nevertheless, these devices are specific medical tools and thus, they are not widely available to a predominantly healthy population with a potential risk for arrhythmia.

Our results showed that assignment errors occurred significantly more often in older subjects and that parameters concerning physical aspects, sex or electrical axis had no impact on ECG assignment (Table 1). Assignment errors in older patients were not caused by worse signal quality but similar ECG morphologies in two leads. Another potential benefit of multichannel ECGs by a smartwatch could be surveillance of sinus rhythm in patients with known or assumed AF if *P* waves are not clearly discriminated in lead I. Based on these findings, ECG recording by the Apple Watch seems to be feasible for a wide range of different types of patients.

Atrial fibrillation is the most common cardiac arrhythmia and responsible for up to 25% of strokes in the US [1]. Furthermore, in 18% of patients with AF-associated stroke, AF is detected only after the stroke event [1]. Recent studies [1,2] showed that detection of AF by using a smartwatch is feasible with very high accuracy. Therefore, increasing use of smartwatches may contribute to enhanced detection of AF by patients themselves, which in turn may improve initiation of adequate oral anticoagulation and consequently reduce AF-associated stroke rates.

All currently published data [1,2,8] used photoplethysmography as a surrogate parameter for the underlying heart rhythm or ECG. In contrast, in our study true bipolar ECG leads were recorded by using a last generation smartwatch. A good ECG signal quality was confirmed, sufficient for diagnostic purposes. Recording of ECGs by a smartwatch might improve arrhythmia diagnostics. Furthermore, smartwatch ECGs may contribute to detection of myocardial ischemia and consequently improve treatment of patients with myocardial infarction. Previous studies demonstrated that only 25% of patients with myocardial infarction contacted the emergency medical service within one hour of symptom onset [9]. Reasons for delay were that symptoms were not recognized as of cardiac origin or that symptoms were not serious [9]. In these patients, use of a smartwatch with ECG recording function might improve detection of myocardial infarction and shorten the time to first medical contact, which might improve patients’ outcome.

Recent studies with smartwatches evaluated primarily their diagnostic role for detection of atrial fibrillation [1,2]. The Apple Heart Study by Turakhia et al. used an Apple Watch with photoplethysmography for identification of pulse irregularity or variability, which may be used for detection of atrial fibrillation (AF) or atrial flutter [1]. The study showed that a smartwatch could reliably detect pulse irregularity and variability which might unmask asymptomatic AF [1,8]. The WATCH AF trial studied diagnostic accuracy of AF detection by a smartwatch-based algorithm using photoplethysmographic signals in comparison with a standard ECG [2]. The authors found that detection of AF using a smartwatch is feasible, with very high diagnostic accuracy [2]. However, applicability of the algorithm was limited due to a high dropout rate based on insufficient signal quality [2].

The study by Tison et al. also demonstrated that smartwatch photoplethysmography was able to detect AF but also found reduced sensitivity and specificity compared to the standard ECG [12]. A limitation of photoplethysmography is that pulse irregularities are used for detection of AF. As supraventricular and ventricular extra systoles may also cause pulse irregularities, AF detection may be impeded by photoplethysmography. Therefore, recording of an ECG by a smart watch may increase diagnostic accuracy. Several handheld electrocardiogram devices are available for recording of a single-lead ECG. The AliveCor device can record Einthoven lead I and was used for community AF screening in a study which found a sensitivity of 98% and specificity of 97% for detection of AF by on optimized algorithm [8]. False positive AF detection was due to small voltage *P* waves in lead I [8]. Another study tested single-lead electrocardiogram (ECG) devices for atrial fibrillation (AF) screening in a hospital population in cardiologic and geriatric patients [4]. The authors demonstrated that sensitivity and specificity of the automated algorithms were suboptimal (Cardiology: 81.8 and 94.2%, respectively, for MyDiagnostick; 54.5 and 97.5%, respectively, for AliveCor; Geriatrics: 89.5 and 95.7%, respectively, for MyDiagnostick; 78.9 and 97.9%, respectively, for AliveCor) and additional 12-lead ECGs are required to optimize specificity. A limitation of these single-lead electrocardiogram (ECG) is that only one ECG lead, mainly Einthoven I, can be recorded.

A study using the single lead ECG device for recording Einthoven lead I showed very high sensitivity of 95–100% and specificity of 90–97% for detection of AF by two cardiologist or an optimized ECG device algorithm [8]. Five false positives in this study were due to small voltage *P* waves in lead I, and two also had multiple atrial ectopics causing irregularity [8]. As this device can record Einthoven lead I only, additional recording of lead II and III might improve ECG quality and increased sensitivity and specificity for AF detection.

## 5. Conclusions

Recording of a three lead ECG with an Apple Watch Series 4 is feasible with good ECG signal quality. Despite the higher placement of the watch in the region of the left lower abdomen, instead of the left lower limb, recorded signals show high consistency to Einthoven leads II and III. Thus, diagnostic potential of the device for detection of arrhythmias and other cardiac diseases can be increased using a simple measurement protocol. This device has the potential to play an important role in detection of arrhythmias and repolarization abnormalities, which might initiate early diagnosis of cardiac disorders by patients themselves.

## 6. Limitations

The four cardiologists performed only visual comparison of the smartwatch ECGs with Einthoven leads I, II and III of the standard ECG. So far, we have no computer-based comparison of accordance of the smartwatch ECGs with the 12-lead ECG. Standard 12-lead ECGs were recorded with a paper running speed of 50 mm/s, whereas smartwatch ECGs were recorded with a paper running speed of 25 mm/s, which might have influenced correct identification of the simulated Einthoven leads. This pilot study was designed as a proof of concept. Therefore, we studied the feasibility of ECG recording by an Apple Watch in healthy subjects. So far, we have no data from patients with heart disease. In a following study, we plan to record smartwatch ECGs in patients with an acute coronary syndrome in order to test feasibility of smartwatch ECG recording and detection of repolarization abnormalities in this subgroup of patients.

## Figures and Tables

**Table 1:** Subject characteristics (BSA = body surface area, BMI = body mass index, HR = heart rate). *P* values were obtained by T-Test and ANOVA; probability limit 95%.

	All	Male	Female	p
**Size (cm)**	174 ± 10	183 ± 8	168 ± 7	<0.001
**Weight (kg)**	78 ± 15	87 ± 16	72 ± 12	0.001
**BSA (m^2^)**	1.84 ± 0.20	2.10 ± 0.16	1.8 ± 0.20	<0.001
**BMI (kg/m^2^)**	25.8 ± 4.8	26.1 ± 4.7	25.6 ± 4.9	0.752
**Age (years)**	40 ± 12	41 ± 9	39 ± 13	0.761
**QRS axis (°)**	48 ± 33	38 ± 38	54 ± 29	0.096
**HR 12 lead ECG (bpm)**	71 ± 13	74 ± 11	69 ± 14	0.234
**HR lead I (bpm)**	71 ± 11	74 ± 10	69 ± 12	0.137
**HR lead II (bpm)**	73 ± 11	76 ± 11	71 ± 11	0.156
**HR lead III (bpm)**	72 ± 12	77 ± 12	70 ± 12	0.060

**Table 2:** Correlations between correct ECG assumption and study population characteristics (BSA = body surface area, BMI = body mass index, HR = heart rate). *P* values were obtained by *t*-test, ANOVA and χ2-test. Probability limit 95%.

	ECG Correct	ECG Incorrect	p
**Size (cm)**	173 ± 9	174 ± 11	0.862
**Weight (kg)**	77 ± 13	80 ± 23	0.486
**BSA (m^2^)**	1.92 ± 0.18	1.96 ± 0.30	0.560
**BMI (kg/m^2^)**	25.6 ± 4.7	26.4 ± 5.3	0.652
**Age (years)**	38 ± 12	46 ± 10	0.032
**QRS axis (°)**	48 ± 35	50 ± 27	0.831
**Sex (m/f)**	13/26	5/6	0.459
**HR 12 lead ECG (bpm)**	71 ± 13	68 ± 11	0.511
**HR lead I (bpm)**	72 ± 12	69 ± 8	0.516
**HR lead II (bpm)**	73 ± 12	71 ± 9	0.740
**HR lead III (bpm)**	73 ± 13	71 ± 9	0.626
